# Implementation of Non-Invasive Quantitative Ultrasound in Clinical Cancer Imaging

**DOI:** 10.3390/cancers14246217

**Published:** 2022-12-16

**Authors:** Deepa Sharma, Laurentius Oscar Osapoetra, Gregory J. Czarnota

**Affiliations:** 1Imaging Research and Physical Sciences, Sunnybrook Health Sciences Centre, Toronto, ON M4N 3M5, Canada; 2Department of Radiation Oncology, Sunnybrook Health Sciences Centre, Toronto, ON M4N 3M5, Canada; 3Departments of Medical Biophysics and Radiation Oncology, University of Toronto, Toronto, ON M5S, Canada

**Keywords:** cell death, locally advanced breast cancer (LABC), treatment response, quantitative ultrasound (QUS), radiotherapy, chemotherapy

## Abstract

**Simple Summary:**

At present, quantitative ultrasound (QUS) is increasingly utilized in cancer imaging. Compared to other imaging modalities that take several days to weeks to assess treatment effectiveness, QUS can provide a rapid treatment evaluation. Its implementation has been documented in characterizing benign versus malignant breast lesions, assessing lymph nodes, predicting and monitoring tumor response, etc. Both preclinical and clinical studies have confirmed that changes in QUS parameters are directly correlated with tissue microstructural alterations. QUS parameters and textural analyses have widely been used to predict and monitor neoadjuvant chemotherapy (NAC) response in locally advanced breast cancer (LABC) patients. Thus, QUS methods have emerged as one of the most useful imaging techniques for the management of several tumor types.

**Abstract:**

Quantitative ultrasound (QUS) is a non-invasive novel technique that allows treatment response monitoring. Studies have shown that QUS backscatter variables strongly correlate with changes observed microscopically. Increases in cell death result in significant alterations in ultrasound backscatter parameters. In particular, the parameters related to scatterer size and scatterer concentration tend to increase in relation to cell death. The use of QUS in monitoring tumor response has been discussed in several preclinical and clinical studies. Most of the preclinical studies have utilized QUS for evaluating cell death response by differentiating between viable cells and dead cells. In addition, clinical studies have incorporated QUS mostly for tissue characterization, including classifying benign versus malignant breast lesions, as well as responder versus non-responder patients. In this review, we highlight some of the important findings of previous preclinical and clinical studies and expand the applicability and therapeutic benefits of QUS in clinical settings. We summarized some recent clinical research advances in ultrasound-based radiomics analysis for monitoring and predicting treatment response and characterizing benign and malignant breast lesions. We also discuss current challenges, limitations, and future prospects of QUS-radiomics.

## 1. Introduction

Over the years, multiple technologies have paved the way in the treatment of cancers. Diagnosing cancer is a multi-step, complex process. Diagnostic imaging procedures have made earlier detection of cancer possible. Some of the commonly used imaging techniques include computed tomography (CT), magnetic resonance imaging (MRI), positron emission tomography (PET), ultrasound, and X-ray [1,2]. Quantitative ultrasound (QUS) is one of such kinds that has gained popularity by offering many advantages over other modalities. It is a non-invasive device that is cost-effective, portable, and radiation-free [3]. No incisions or cuts are required during the imaging process, which makes it a safe and easily accessible device. The use of QUS has widely been explored both as a diagnostic and therapeutic imaging modality.

Several preclinical and clinical studies have implemented QUS for cancer prediction and monitoring [4,5,6,7,8,9]. Different parameters can be extracted from linear regression analysis using normalized power spectra of radio frequency (RF) data from ultrasound [10,11,12,13,14,15]. Linear-fit parameters include the mid-band fit (MBF), 0-MHz intercept (SI), and spectral slope (SS). The first two parameters are associated with the amount of backscattering, while the last one is associated with the scatter’s size [10,11,12]. In order to obtain spectral parameters that reflect more about acoustic scatterers, such as their concentration and size, theoretical acoustic scattering models can be fitted to the measured back-scattering function of the sample. This results in average scatterer diameter (ASD)/effective scatterer diameter (ESD)/effective scatterer size (ESS) and average acoustic concentration (AAC)/effective acoustic concentration (EAC)/effective scatterer concentration (ESC) parameters. These parameters (ASD and AAC) can be estimated using the spherical Gaussian scattering model (SGM) and the fluid-filled-sphere model (FFSM) form factor models to the ultrasonic backscatter coefficient (BSC) [13,14,15]. The ASD and AAC parameters correlate with the changes in scatterer sizes and concentrations, respectively. QUS spectral parametric imaging creates maps of quantitative parameters obtained through spectral analysis of ultrasound RF data. These maps reveal tissue intrinsic scattering properties. Tissue microstructures and their scattering properties are distinct between invasive and non-invasive tumors [16], and from responsive to treatment compared to those of non-responsive ones [17]. The QUS spectral parametric images can provide a surrogate delineation of tumor microstructures providing diagnostic [18,19] and prognostic potentials [20,21,22,23,24,25]. QUS spectral parametric maps are created using a sliding window technique. The window extracts a block of RF data for spectral analysis. The kernel is moved to all points in the region of interest (ROI), given some kernel overlap factors. For clinical applications, typically a 2 mm × 2 mm kernel size is used. This size amounts to approximately ten acoustic wavelengths. A Hanning gating function is applied in the axial direction to smooth out the RF segments. The Fast Fourier Transform (FFT) method is utilized to extract the spectral contents of each RF segment in the block. An average power spectrum associated with the kernel is then obtained by averaging RF spectra in the kernel.

A reference phantom technique is utilized to obtain tissue-dependent scattering components from the RF signal [26,27]. The spatially homogeneous reference phantom has well-characterized frequency-dependent back-scattering and attenuation functions. The phantom is scanned using the same ultrasound system and acquisition settings for extracting reference RF data. Corresponding to the RF block from the sample, the reference RF block is acquired from the same location in the reference phantom. The spectral normalization procedure divides the sample spectrum by the reference spectrum. Along with the corresponding attenuation functions and reference backscatter coefficient (BSC), this allows for the estimation of the tissue-dependent scattering function. Parametrization of the frequency-dependent scattering function results in the linear-fit and acoustic scattering parameters useful for diagnostics and prognostics [18,19,20,21,22,23,24,25].

**Figure 1 cancers-14-06217-f001:**
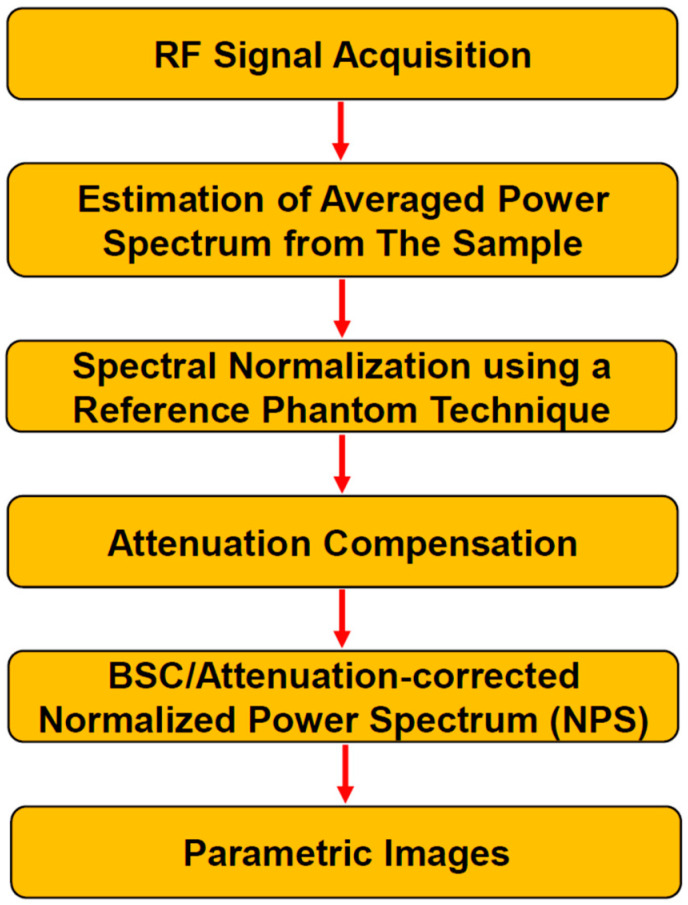
A summary of QUS spectroscopy workflow. QUS spectroscopy extracts scattering components from the RF signal that uniquely characterize tissue samples. The spectral normalization procedure removes instrument-dependent components from the RF signal, while the attenuation correction procedure compensates loss in the acoustic signal as it propagates through intervening tissue layers. Parametrization of the attenuation-corrected NPS or the BSC allows for tumors characterization, providing diagnostic and prognostic values. These parameters can then be utilized to develop a classification model for diagnostic and prognostic purposes. BSC: backscatter coefficient, NPS: normalized power spectrum, QUS: quantitative ultrasound.

These analyses are repeated for each point in the ROI to come up with parametric images of QUS spectral parameters. From the parametric maps, radiomics features can be extracted that include first-order statistical, morphological, and textural features. These radiomic features are potential imaging biomarkers for diagnosis and prognosis purposes [18,19,20,21,22,23,24,25]. Radiomics texture features rely on the hypothesis that tissue heterogeneity can be quantified through their surrogate spectral parametric maps (Figure 1).

### 1.1. Ultrasound Imaging of Cell Death in Tumor Response

Studies have confirmed a strong correlation between changes in cell nuclear structure and ultrasound parameters using both low- and high-frequency ultrasound. A study by Banihashemi et al. showed an increase in MBF and SS parameters in a time-dependent manner following photodynamic therapy (PDT) in an in vivo melanoma model. They reported 45% apoptotic cell death at 24 h, that corresponded to maximum SS increase, showing a value of 0.435 ± 0.07 dB/MHz. Similarly, an increase in MBF was observed between 12 to 24 h. At 48 h, around 50% of the cells were seen to have their nuclei disappeared as a result of late stage of apoptosis. The loss of cell nuclei at 48 h resulted in the decrement of ultrasound backscatter parameters [28]. Thus, a direct correlation between cell death and ultrasound parameters was established in this study. A similar outcomes were reported by Sadeghi-Naini et al. in a clinical study conducted with locally advanced breast cancer (LABC) patients receiving chemotherapy. They reported an increase in MBF and SI at week 4 of treatment. Responding patients demonstrated 9.1 ± 1.2 dBr increases in MBF as compared to the non-responder with the MBF value of 1.9 ± 1.1 dBr. Similarly, the SI variable increased in responding patients to 8.9 ± 1.9 dBr compared to 1.6 ± 0.9 dBr observed in non-responding patients. Additionally, histopathology data from the non-responder patient demonstrated a large compact mass in the mastectomy specimen; however, no such mass was observed in the specimen of responder patients. Thus, their result showed that patients responding to treatment showed significant changes in QUS parameters as compared to non-responders [5]. Moreover, parameters such as AAC and ASD have also been found to strongly correspond to cell death. A clinical study reported a substantial increase in AAC and ASD at weeks 1, 4, and 8 in LABC patients that responded to chemotherapy. Maximum increase was observed at week 8. On the contrary, no such increase was seen in non-responding patients [13]. Similar results were also reported with breast cancer xenograft following an exposure to chemotherapy. Higher AAC corresponded to the highest cell death, indicating 60% at 24 h. A strong correlation between AAC and cell death was demonstrated with (*R*^2^ SGM = 0.40) [14]. Thus, many endeavors have been made that confirm the utility of QUS and its parameters for monitoring treatment response, as well as for differentiating between clinically responding and non-responding patients. In the following sections, we discuss in detail some of the important findings that used QUS in clinical settings for cancer diagnosis, tissue characterization, treatment prediction and monitoring. The summary of QUS application and its utilization in preclinical and clinical studies is presented in Table 1.

### 1.2. Clinical Applications of Classification Models Developed from QUS Spectral Parametric Images Using Machine Learning Approaches

#### Tumors Characterization

Apart from monitoring tumor response and treatment efficacy, the use of QUS in classifying benign versus malignant tumors has also been widely studied [54,61,62]. The first study by Sadeghi-Naini et al. utilized QUS spectral and textural analysis techniques to characterize breast lesions from 78 patients. Intra-lesion heterogeneity within tissue micro-structures was quantified using textural features and average-based mean-value parameters from each parametric map [54]. Later on, Osapoetra et al. expanded this study to a larger cohort by analyzing texture and texture-derivate within the peri-tumoral breast tissue [61]. Both of the studies only utilized the Gray Level Co-Occurrence Matrix (GLCM)-based texture method to characterize breast lesions. Furthermore, another study by Osapoetra et al. incorporated different texture methods, including GLCM, the Gray Level Run Length Matrix (GLRLM), and the Gray Level Size Zone Matrix (GLSZM). They applied these methods to QUS spectral parametric images to characterize breast lesions from the tumor core and tumor margin [62].

A study by Sadeghi-Naini et al. explored different texture features to discriminate between benign versus malignant breast lesions. Based on the size, density, and distribution of acoustic scatterers, the QUS texture parameters can be utilized to quantify intra-lesional heterogeneity, providing characterization and estimation of tissue microstructure. In their study, several texture features, including contrast (CON), correlation (COR), homogeneity (HOM), and energy (ENE), were generated using parametric maps of MBF, SS, SI, SAS, EAC, and ESD. These texture features were then used as biomarkers to classify breast lesions. The result revealed significant differences in benign versus malignant lesions with (*p* < 0.05) in most of the textural features, including MBF (CON), (COR), (HOM), SS (CON), (COR), (HOM), and SI (CON), (COR), (HOM), SAS (ENE), ESD (CON), (COR), and EAC (HOM), (ENE) [54].

**Figure 2 cancers-14-06217-f002:**
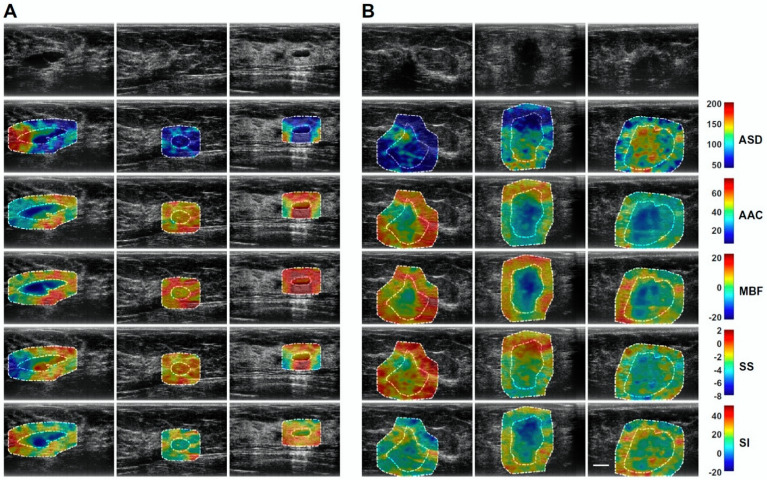
Representative QUS spectral parametric images from benign and malignant group. (**A**) benign breast lesions (left columns) (**B**) malignant breast lesions (right columns). The color bars present the range for ASD of 160 μm, AAC of 70 dB/cm^3^, MBF of 44 dB, SS of 10 dB/MHz, and SI of 70 dB. Scale bar: 1 cm. ASD: average scatterer diameter, AAC: average acoustic concentration, MBF: mid-band fit, SS: spectral slope, SI: 0-MHz intercept. Reprinted with permission from: (Figure 1) [61].

**Figure 3 cancers-14-06217-f003:**
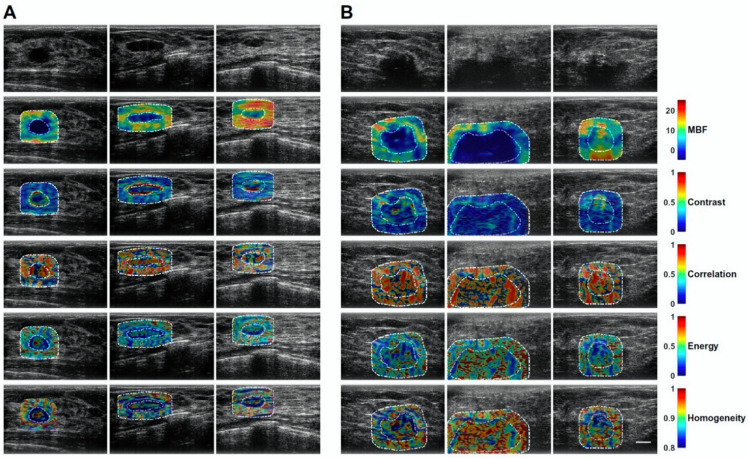
Representative texture maps representing local quantification of image texture. (**A**) benign breast lesions (left columns) (**B**) malignant breast lesions (right columns). Scale bar: 1 cm. MBF: mid-band fit. Reprinted with permission from: (Figure 2) [61].

**Figure 4 cancers-14-06217-f004:**
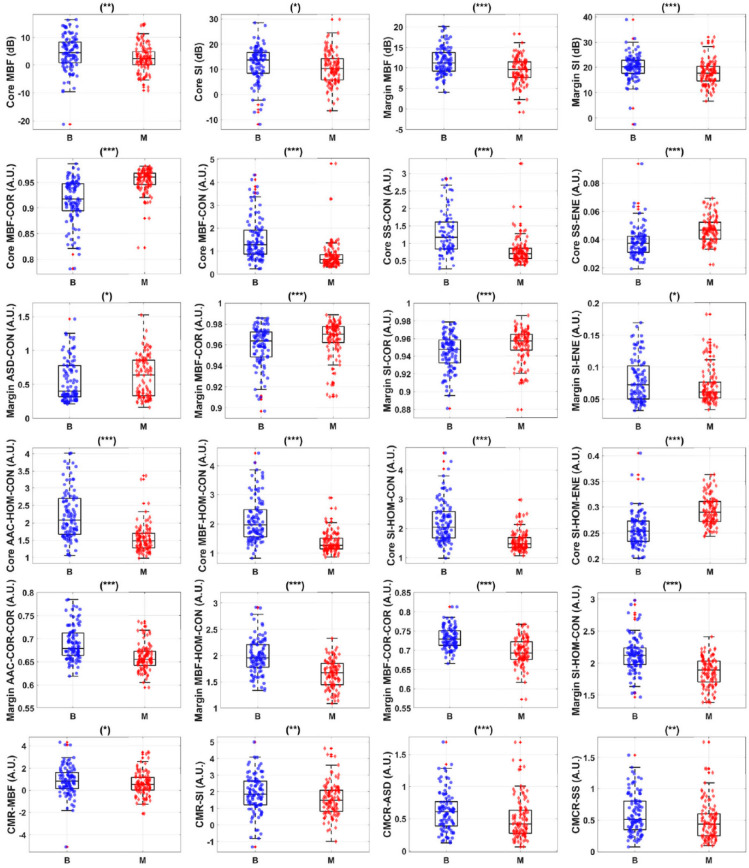
Box and scatter plots of representative radiomics features that demonstrate statistically significant differences. Statistical significant, are represented as (* *p* < 0.05), (** *p* < 0.01), and (*** *p* < 0.001). B: benign breast lesions, M: malignant breast lesions, ASD: average scatterer diameter, AAC: average acoustic concentration, MBF: mid-band fit, SS: spectral slope, SI: 0-MHz intercept, CON: contrast, COR: correlation, ENE: energy, HOM: homogeneity. Reprinted with permission from: (Figure 3) [61].

QUS spectral parametric imaging and texture analyses have been applied for the characterization of breast lesions [61,62]. In this task, Osapoetra et al. developed classification models using radiomics features extracted from the parametric images, along with traditional machine learning classifiers, to distinguish malignant lesions from benign breast lesions [61]. In that study, the authors hypothesized that texture-derivate features exhibit significant discriminating power for developing a multi-feature classification model. Their cohort consisted of 204 patients with breast lesions (99 benign and 105 malignant). Figure 2 and Figure 3 show representative B-mode, ASD, AAC, MBF, SS, and SI parametric maps from the benign and malignant groups, respectively. Figure 4 depicts scatter and box plots of representative discriminating features. Texture-derivate features were demonstrated as ones with the most separation between the two groups. They reported the best classification performance of 90% sensitivity, 92% specificity, 91% accuracy, and 0.93 area under the curve (AUC) using features from the tumor core and margin [61].

In another study, different ways to quantify texture images were studied for the task of characterizing breast lesions. Classification models were developed using the GLCM, GLRLM, and GLSZM radiomics features of QUS spectral parametric images. They reported a classification accuracy from 73–91%, depending on tumor and margin attributes and classification algorithms, on a cohort of 193 patients with breast lesions (92 benign and 101 malignant cases) [62].

QUS spectroscopy have also been applied for the characterization of different types of cancer. Rohrbach et al. demonstrated an application of QUS in the non-invasive characterization of prostate cancers using a novel transrectal high-frequency ultrasound system (ExactVu micro-ultrasound Exact Imaging, Markham, ON, Canada). They developed a multi-variate model from ASD, AAC, envelope statistics, and prostate-specific antigen (PSA) features, achieving the best AUC of 0.81 using a linear discriminant classifier [55]. Furthermore, Goundan et al. reported a thyroid nodules (TNs) characterization study [63]. The cohort consisted of 225 TNs (24 malignant and 201 benign) from 208 patients. They developed a classification model using QUS spectral features that include ASD, AAC, MBF, and SI parameters, along with an envelope statistics feature in the Nakagami shape parameter. Their model achieved the best classification performance of 0.857 ± 0.033 receiver operating characteristics (ROC) AUC, which was comparable with the value achieved using the American College of Radiology Thyroid Imaging, Reporting and Data System (ACR TI-RADS) and American Thyroid Association (ATA) risk-stratification systems. Furthermore, they also demonstrated a reduction in the number of unnecessary fine-needle biopsies (FNBs) when the combination of QUS parameters were used separately or with the ACR TI-RADS [63].

### 1.3. LABC QUS Treatment Response Prediction

QUS spectral parametric imaging and texture analyses have been applied for the clinical application of response characterization to cancer treatments. The underlying hypothesis is that QUS spectral parameters are sensitive enough to detect microscopical changes in tumors. As the tumors respond to cancer therapeutics, these changes can be used as imaging biomarkers for developing classification models. Such models are potentially useful in clinical practice, as they can guide early interventions during the full course of cancer treatment. For example, in the neoadjuvant chemotherapy (NAC) management of LABC, early prediction of non-responder patients will allow clinicians to decide if other therapeutic agents might fare better for the objective of reducing the tumor size prior to the main surgical procedure to remove the LABC tumors.

LABC treatment response prediction models from QUS spectral parametric imaging and texture methods have been reported in a number of recent studies [20,21,22,23,25]. These studies investigated several aspects of the classification model for treatment response prediction that utilize radiomics features from higher-order GLCM-based textural features [21,24]; assessment of potential issues regarding the variability of the radiomics features as a result of different ultrasound systems in the clinic [20]; and model evaluation from multi-institutional data [18,21], utilization of a novel approach that extract radiomics features from intra-tumoral regions obtained through an unsupervised segmentation technique [22], and development of a deep learning-based model [23].

First, Dasgupta et al. studied the classification performance of models developed utilizing higher-order textural features (texture-derivative) [58]. The cohort consisted of 100 patients (83 responders and 17 non-responders) with LABC undergoing NAC. They extracted a pool of radiomics features, including the first-order statistical mean-value, GLCM-based texture, and the proposed GLCM-based texture-derivate features. The best set of features was determined through a sequential feature selection approach to develop classification models to identify non-responders from responders. They reported the best classification performance of 87% sensitivity, 81% specificity, 82% accuracy, and 0.86 AUC [58].

**Figure 5 cancers-14-06217-f005:**
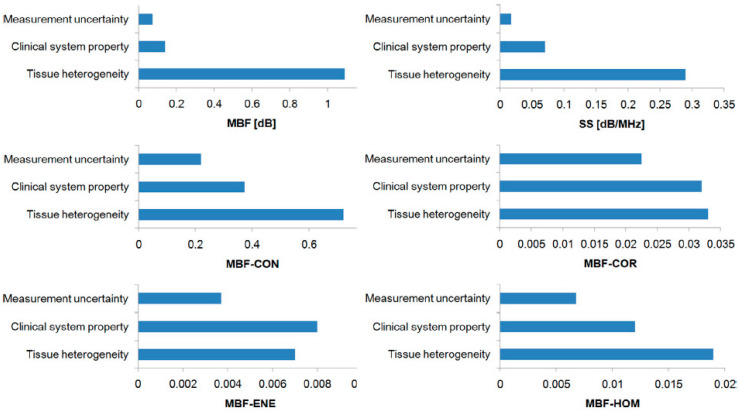
The root means square deviation of differences in QUS parameters: MBF and SS, MBF-texture parameters: MBF-CON, MBF-COR, MBF-ENE, and MBF-HOM due to measurement uncertainty, variations in ultrasound systems, and tissue heterogeneity. These results suggest that the inherent tissue heterogeneity was the most dominant contributor to variations in the estimated radiomics features of QUS spectral parametric images. Overall, measurement uncertainty and variations in clinical ultrasound systems contribute less than the tissue heterogeneity component. MBF: mid-band fit, SS: spectral slope, CON: contrast, COR: correlation, ENE: energy, HOM: homogeneity. Reprinted with permission from: (Figure 3) [20].

**Figure 6 cancers-14-06217-f006:**
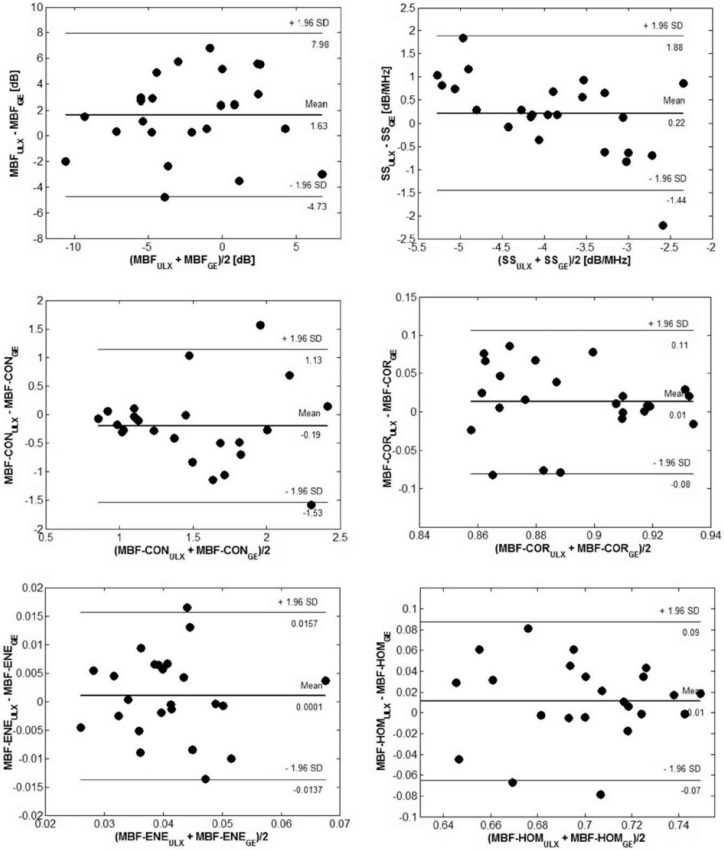
Bland-Altman plots comparing radiomics features obtained using an Ultrasonix-RP (Ultrasonix Medical Corp., Richmond, BC, Canada) and a GEGE-LOGIQ E9 (General Electric Healthcare, Milwaukee, WI, USA) ultrasound system. The limits of agreement were indicated by the mean difference and ± standard deviation. MBF: mid-band fit, SS: spectral slope, CON: contrast, COR: correlation, ENE: energy, HOM: homogeneity, ULX: ultrasonix RP L14-5/60, GE: GE-LOGIQ 9 L-D. Reprinted with permission from: (Figure 6) [20].

Second, Sannachi et al. addressed the potential issue of variations in the measured radiomics features as they were acquired across different ultrasound systems prevalent in clinics, along with their impacts on model classification performance. A GE-LOGIQ E9 (General Electric Healthcare, Milwaukee, WI, USA) and Ultrasonix-RP ((ULX) Ultrasonix Medical Corp., Richmond, BC, Canada) ultrasound system were utilized. They analyzed radiomics features from a cohort of 24 patients with LABC [20]. A previously developed classification model for treatment response monitoring was also applied to assess the performance of the smaller cohort [54]. They concluded that observed variations in data due to system-specific features were small, and the results of the prediction models were comparable across both ultrasound systems utilized Table 2. They found tissue heterogeneity to be the dominant factor causing variations in the measured radiomics features [20] (Figure 5 and Figure 6).

DiCenzo et al. studied the efficacy of the classification model for therapy response prediction using LABC data from multiple institutions. Their cohort consisted of 82 patients with LABC undergoing NAC (48 responders and 34 non-responders). The patients included were from multiple institutions. They developed classification models using a pool of pre-treatment radiomics features that include first-order statistical mean-value and GLCM-based texture features. They reported the best performance metric of 87% accuracy in predicting non-responders from responders [21]. Subsequently, Osapoetra et al. also assessed the classification model for therapy response prediction, utilizing more pre-treatment radiomics features that include higher-order GLCM-based texture on a similar multi-institutional dataset. The cohort consisted of 74 patients (42 responders and 32 non-responders) with LABC. Higher-order texture features have shown their discriminating power for breast lesions characterization datasets. They reported the best classification performance of 88% sensitivity, 78% specificity, 84% accuracy, and 0.86 AUC [18].

In the typical framework for building a classification model for treatment response prediction using radiomics texture features, the average value or average texture features were estimated from the whole QUS spectral parametric image. Taleghamar et al. proposed a different approach to utilizing spatial information from intra-tumoral parametric maps. Their approach first segmented a discrete number of regions of the tumor from the parametric maps, prior to obtaining average features. They utilized an unsupervised learning approach for segmentation using a hidden Markov random field (HMRF) expectation maximization (EM) algorithm. Their approach allows for the utilization of radiomics features only from a certain region of the intra-tumoral parametric maps. Their cohort consisted of 181 patients (138 responders and 43 non-responders) with LABC undergoing NAC. The radiomics features included first-order statistical mean-value and signal to noise ratio (SNR) from each QUS spectral parametric image at pre-treatment. They reported a classification model using the Adaptive Boosting (AdaBoost) technique, attaining 85.4% accuracy and 0.89 AUC on an independent test set [22].

Deep learning models learn how to represent data with multiple levels of abstraction using multiple processing layers [64]. They learn the features directly from data, in contrast to traditional machine learning involving feature engineering. These methods have set the state-of-the-art performance for tasks in computer vision, natural language processing, and many other domains [39]. This trend motivated a recent effort by Taleghamar et al. in utilizing the deep learning framework to boost the performance of the classification model for treatment response prediction [23]. In contrast to previous studies on deep learning for breast lesions characterization that use B-mode images, the inputs to the network are QUS spectral parametric images [22]. In that study, Taleghamar et al. implemented convolutional neural network (CNN) models for treatment response prediction [23]. Their approach utilized a modified residual network version 101 (ResNet) [65] and a modified residual attention network version 56 (RAN) [66] as the convolutional base. A densely connected classifier was attached on top of the base layer. The networks were trained from scratch using augmented QUS spectral parametric images from their cohort. The cohort consisted of 181 LABC patients (138 responders and 43 non-responders) undergoing NAC. They reported the best performance of 88% accuracy and 0.86 AUC on an independent test set [23].

### 1.4. Head and Neck QUS Treatment Response Prediction

**Figure 7 cancers-14-06217-f007:**
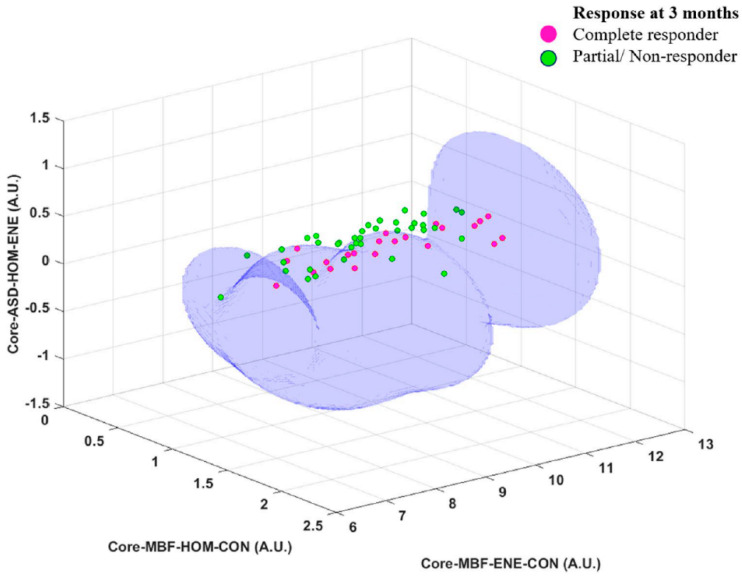
A representative nonlinear decision boundary obtained from an SVM model using the RBF kernel to separate complete responders from those with partial/non-responders. The model consisted of the best combination of three radiomics features obtained through a forward sequential feature selection with cross-validation. SVM: support vector machines, RBF: radial basis functions, MBF: mid-band fit, CON: contrast, ENE: energy, HOM: homogeneity. Reprinted with permission from: (Figure 3) [19].

Another application of QUS radiomics was for building classification models to predict treatment response in 59 patients (22 early responders (ER), 29 late-responders (LR), and 8 progressive diseases (PD)) with head and neck squamous cell carcinoma (HNSCC) undergoing radical radiotherapy (RT) with or without concurrent chemotherapy. The classification tasks identified non-responder (NR) from responders (R). Furthermore, they also separated NR into LR and those with PD [19]. Table 3 tabulates the classification metrics in the classification of ER from partial or NR. Table 4 summarizes the classification performance in the classification of LR versus persistent or PD. Figure 7 depicts a representative decision boundary, along with points from the two groups in a three-dimensional space of the selected best three features [19].

Tran et al. [57] reported classification models for predicting treatment response to radical RT in patients with head and neck malignancies. Their cohort consisted of 36 patients, with 14 complete responders (CR) and 22 partial responders (PR). Classification models were built using the best combination of attributes from a pool of radiomics features. The radiomics features consisted of first-order statistical mean-value and GLCM-based texture features of QUS spectral parametric images obtained at 24 h, 1 week, and 4 weeks post-treatment. They reported the best classification accuracies of 80, 86, and 85% at 24 h, week 1, and week 4, respectively [57].

## 2. Recurrence Prediction

### 2.1. Recurrence Prediction of Head and Neck Cancer Using Radiomics of QUS Spectral Parametric Imaging

Radiomics of QUS spectral parametric imaging have been applied for a clinical application in the identification of recurrence in 51 patients (17 recurrences vs. 34 non-recurrence) with head-neck squamous cell carcinoma (HNSCC) treated with RT with or without concurrent chemotherapy [24,59]. Dasgupta et al. implemented traditional machine learning in building a classification model for recurrence prediction. A set of pre-treatment radiomics features that included first-order statistics mean-value and GLCM-based textures were extracted from the parametric images. They reported the highest classification performance of 76% sensitivity, 71% specificity, 75% accuracy, and 0.74 AUC with the KNN (k-nearest neighbors) classifier [24].

Fatima et al. presented a radiomics study that develop a classification model for predicting recurrence in a cohort of 51 patients (17 recurrences vs. 34 non-recurrence) with HNSCC. In that study, radiomics imaging features from QUS spectral parametric images included mean-value first-order statistics and GLCM-based textures. Radiomics features from pre-treatment were subtracted from those collected at week 1 and week 4 after starting RT. They developed classification models using delta-features and reported classification performance of 80% and 82% accuracy in predicting recurrence using changes in the radiomics features at week 1 and week 4 of therapy, respectively [59].

### 2.2. Recurrence Prediction of Locally-Advanced Breast Cancer Using Radiomics of QUS Spectral Parametric Imaging

Dasgupta et al. reported a classification model to predict recurrence using radiomics features of QUS spectral parametric images obtained at pre-treatment [25]. Their cohort consisted of 83 patients (28 recurrences versus 55 non-recurrence) with LABC undergoing NAC. The pool of radiomics features consisted of first-order statistical mean-value, GLCM-based texture, and GLCM-based higher-order textures. They reported the best classification performance of 71% sensitivity, 87% specificity, 82% accuracy, and 0.76 AUC using an support vector machine (SVM) classifier. Using the same data, they also evaluated different targets in predicting recurrence. They reported a five-year recurrence-free survival of 83% and 54% (*p* = 0.003) and a five-year overall survival of 85% and 74% (*p* = 0.083) using the SVM model [25].

Bhardwaj et al. developed a classification model for predicting disease relapses in a cohort of 83 patients with LABC (28 recurrences and 55 non-recurrence) treated with NAC [60]. The pool of radiomics features included first-order statistical mean-value, GLCM-based textures, and GLCM-based higher-order textures from QUS spectral parametric images. They reported the best model from baseline and changes in week 4 features, achieving 87% sensitivity, 75% specificity, 81% accuracy, and 0.83 AUC [60].

## 3. Conclusions

In summary, QUS and its spectroscopic variables can be used to monitor treatment response, as well as to characterize responders versus non-responders and benign versus malignant tumor lesions and so on. Along with this, QUS is one of the fastest standard methods to predict treatment outcome. While most of the traditional methods incorporate tumor size measurement as a gold standard for response monitoring, that takes up to several days to weeks or even months. In contrast, imaging techniques such as QUS can provide details of tissue morphological alterations within a few hours. Thus, understanding the tumor heterogeneity and treatment response using QUS in advance might allow treatment switch if necessary at the earliest possible time.

Application of radiomics to QUS spectral parametric imaging has found a plethora of utilization in tumors characterization, treatment response characterization, monitoring, and prediction of disease relapses. Despite these promising avenues, caution needs to be exercised in regards to its implementation. These studies rely on the concept of learning from labeled data and the evaluation of generalized performance on new unseen data. Ongoing efforts are being made to refine the model building and evaluation strategies in order to eliminate potential pitfalls related to leakage of information from training to validation or test sets.

Radiomics features of QUS spectral parametric images are potential attributes for developing robust classification models for computer-aided diagnosis (CAD) systems with immense potential for its future incorporation clinically to evaluate cancer treatment responses.

### Limitations and Future Directions

There are potential limitations associated with the usage of QUS. Since ultrasound is a handheld device, its reproducibility remains a question as compared to other techniques, such as mammography or MRI, that are automatic with high accuracy. Another important point is that most of the studies conducted using QUS are retrospectively performed at a single center, which suggests that the model might not be fully stable and there might also be a lack of robustness regarding the system. Although normalization and attenuation correction procedures compensate for instrument-specific components in the RF signal, there is a need to perform standardized measurements across institutions, clinical trials, and ultrasound system vendors. Such studies will corroborate the reproducibility and conformity of QUS backscatter measurements across possible sources of variations. Anderson et al. reported an interlaboratory study that independently measured ultrasound BSC from several well-characterized phantom materials [43]. In that study, the phantom materials were constructed with known scatterers’ distribution, allowing a direct comparison of the estimated BSCs with those obtained from acoustic scattering theory. The phantoms were grouped into low-attenuating and tissue-like attenuating materials. Ultrasonic backscattering characterizations of these phantoms were performed over frequency analysis ranging from 1 to 12 MHz. Each institution utilized several ultrasonic transducers with different characteristics, including nominal center frequencies, analysis bandwidths, aperture numbers, and focal lengths. The authors observed an excellent agreement between the BSC estimates from both laboratories. Their study demonstrates the ability to accurately estimate parameters derived from the BSC, including linear fit parameters, along with the scattering parameters. Thus, more inter-institutional studies should be conducted, which not only will provide large sample sizes, but also provide a variety of datasets that can improve the accuracy of the models.

## References

## Figures and Tables

**Table 1 cancers-14-06217-t001:** Summary of QUS application and utilization in preclinical and clinical studies.

QUS Frequency	Implementation	References
50 MHz	Cell death characterization (in vitro)	[29]
40 MHz	Monitoring treatment response (in vitro, in situ and in vivo)	[30]
7 MHz	Tissue characterization (clinical)	[31]
5.75 MHz	Tissue characterization (clinical)	[32]
30 MHz and 34 MHz	Cell death characterization (in vitro)	[33]
4–12 MHz	Tissue characterization (in vivo)	[34]
8 MHz	Tissue characterization (in vivo)	[35]
8.5 MHz and 20 MHz	Tissue characterization (in vivo)	[36]
7.5 MHz	Tissue characterization (clinical)	[37]
40 MHz	Tissue characterization (in vivo)	[38]
20 MHz	Examining cell structural changes (in vitro)	[39]
20 MHz	Tissue characterization (in vivo)	[40]
26 MHz	Monitoring treatment response (in vivo)	[28]
20 MHz	Monitoring treatment response (in vitro)	[41]
20 MHz	Monitoring treatment response (in vivo)	[42]
1–12 MHz	Tissue characterization (phantoms) (clinical)	[43]
25.6 MHz	Lymph nodes characterization (clinical)	[44]
25.6 MHz	Lymph nodes characterization (clinical)	[45]
25 MHz	Monitoring treatment response (in vivo)	[46]
10 MHz and 15 MHz	Tissue characterization (phantoms) (clinical)	[47]
7 MHz	Monitoring treatment response (clinical)	[5]
25.6 MHz	Detecting lymph node metastases (clinical)	[48]
∼7 MHz and 20 MHz	Treatment response monitoring (in vivo)	[4]
40 MHz	Tissue characterization (in vivo)	[49]
6 MHz	Tissue characterization (clinical)	[50]
25 MHz	Monitoring treatment response (in vivo)	[6]
6 MHz	Monitoring treatment response (in situ)	[51]
7 MHz	Monitoring treatment response (clinical)	[13]
~7 MHz	Monitoring treatment response (in vitro, in vivo)	[7]
7 MHz and 20 MHz	Monitoring treatment response (in vivo)	[14]
6 MHz	Monitoring treatment response (in vivo)	[52]
25 MHz	Cell death characterization (in vitro)	[53]
25 MHz	Monitoring treatment response (in vivo)	[8]
~6 MHz	Tissue characterization (clinical)	[54]
29 MHz	Tissue characterization (clinical)	[55]
6.5 MHz and 6.9 MHz	Predicting treatment response (clinical)	[21]
6.3 MHz and 7 MHz	Monitoring treatment response (clinical)	[56]
8 MHz	Monitoring treatment response (clinical)	[57]
7 MHz	Predicting treatment response (clinical)	[58]
6.5 MHz	Predicting tumor recurrence (clinical)	[59]
6.5 MHz	Predicting treatment response (clinical)	[19]
7 MHz	Predicting tumor recurrence (clinical)	[25]
7 MHz	Predicting tumor recurrence (clinical)	[60]

**Table 2 cancers-14-06217-t002:** Summary of classification performance in the classification of non-responder from responder at weeks 1, 4, and 8 after the initiation of chemotherapy using data from two ultrasound systems. The asterisk (*) represents a statistically significant difference (*p* < 0.05) carried between treatment response predictions from two ultrasound systems. The classification model for treatment response monitoring was based on a previously reported study. US: ultrasound, ULX: ULX L14- 5/60, GE: GE-LOGIQ E9. Adapted with permission from [20].

Scan Time	US System	Sensitivity [%]	Specificity [%]	Accuracy [%]	McNemar *p* *
Week 1	ULX	60.0	50.0	58.8	0.752
	GE	60.0	50.0	58.8	
Week 4	ULX	78.9	66.7	77.3	0.545
	GE	64.3	66.7	69.1	
Week 8	ULX	71.4	100.0	73.3	0.683
	GE	71.4	100.0	73.3	

**Table 3 cancers-14-06217-t003:** Response at 3 months: Complete responder (early responder) (*n* = 22) vs. partial/non-responder (*n* = 37). Representative performance of classification models for identifying complete responders from partial or non-responder. The analysis indicated the SVM-RBF model performed the best in this particular task. FLD: Fischer’s linear discriminant analysis, KNN: k-nearest neighbour, SVM-RBF: support vector machine-radial based function, MBF: mid-band fit, SI: 0-MHz intercept, SS: spectral slope, ASD: average scatterer diameter, AAC: average acoustic concentration, COR: correlation, CON: contrast, HOM: homogeneity, ENE: energy. Adapted with permission from [19].

Classifier	Sensitivity	Specificity	Accuracy	AUC	Selected Features
FLD	73	81	78	0.75	MBF-HOM-CON, MBF, SI-CON-ENE
KNN	73	84	80	0.80	SS-COR-COR, MBF-ENE-HOM
SVM-RBF	86	95	92	0.91	MBF-HOM-CON, MBF-ENE-CON, ASD-HOM-ENE

**Table 4 cancers-14-06217-t004:** Final Response. Late responder (*n* = 29) vs. persistent/progressive disease (*n* = 8). FLD: Fischer’s linear discriminant analysis, KNN: k-nearest neighbour, SVM-RBF: support vector machine-radial based function, MBF: mid-band fit, SI: 0-MHz intercept, SS: spectral slope, ASD: average scatterer diameter, AAC: average acoustic concentration, CON: contrast, COR: correlation, ENE: energy, HOM: homogeneity. Adapted with permission from [19].

Classifier	Sensitivity	Specificity	Accuracy	AUC	Selected Features
FLD	86	100	89	0.92	AAC-ENE-HOM, AAC-HOM-CON
KNN	93	88	92	0.90	AAC-HOM, ASD-ENE-HOM
SVM-RBF	97	88	95	0.97	SS, SS-HOM-CON
